# Physicians’ experiences with pharmacists as new members of the interprofessional emergency department team. A qualitative study

**DOI:** 10.1371/journal.pone.0317298

**Published:** 2025-01-13

**Authors:** Tine Johnsgård, Renate Elenjord, Birgitte Zahl-Holmstad, Kristian Svendsen, Elin C. Lehnbom, Eirik H. Ofstad, Torsten Risør, Beate H. Garcia

**Affiliations:** 1 Hospital Pharmacy of North Norway Trust, Tromsø, Norway; 2 Department of Pharmacy, Faculty of Health Sciences, UiT the Arctic University of Norway, Tromsø, Norway; 3 Department of Community Medicine, Faculty of Health Sciences, UiT the Arctic University of Norway, Tromsø, Norway; 4 Department of Medicine, Nordland Hospital Trust, Bodø, Norway; 5 Department of Public Health, Faculty of Health and Medical Sciences, University of Copenhagen, Copenhagen, Denmark; Ataturk University, Faculty of Pharmacy, TÜRKIYE

## Abstract

Pharmacists in emergency departments (EDs) can alleviate physicians’ workload by handling medication-related tasks and offer valuable contributions in interprofessional teams. We aimed to explore physicians’ experiences working with pharmacists in EDs, and their perspectives on future permanent collaboration. We conducted semi-structured interviews with twenty physicians from two EDs and analyzed the data using thematic analysis. Four main themes emerged, comprising twelve subthemes that highlighted both challenges and motivations. *Theme 1*: *time* addressed physicians’ time constraints, and the potential for time reallocation with assistance from pharmacists. *Theme 2*: *various roles of pharmacists* focused on the diverse roles of pharmacists who supported patient care and junior physicians, but faced challenges like availability and space constraints. *Theme 3*: *teamwork* concerned how pharmacists were trusted, brought valuable insights, and enhanced patient safety, yet there were ambiguous views on responsibility and cultural differences. *Theme 4*: *future perspectives* focused on how physicians favored a permanent inclusion of pharmacists in the ED, suggesting that they could independently conduct MedRec. Our findings suggest that pharmacists should be permanently integrated in ED teams. However, there is a need to address challenges related to space and legal regulations to enhance interprofessional collaboration.

## Introduction

Medication errors and medication-related problems place significant burden on both patients and healthcare systems [1]. Suboptimal therapy, incorrect medication use, and medication errors are frequently related to emergency department (ED) visits [2]. Medication errors occur in up to 60% of ED patients [3, 4], while medication discrepancies are reported in as many as 80% of hospitalized patients [5]. Medication reconciliation (MedRec) and medication review are crucial tools for identifying these problems [3, 6]. Detecting and resolving medication-related problems in the ED can prevent them from occurring after patients are transferred to the next level of care. However, ED physicians have in previous studies reported that they do not always have the necessary time to perform these tasks in the ED and that patients can be hospitalized with some uncertainties concerning the medication list [7, 8].

Pharmacists can play a pivotal role in healthcare services, ensuring safe and effective drug regimens, monitoring for adverse effects, and providing valuable contributions into interprofessional teams [9]. Their contributions can lead to better patient outcomes, reduced medication errors, and cost-effective treatment, ultimately improving the quality of healthcare services [10, 11]. In countries like the US and the UK, hospital and primary care pharmacists play crucial roles in patient follow-up. This collaborative practice effectively lightens the burden on other healthcare professionals (HCP) and has positive patient- and healthcare utilization outcomes [12, 13]. Even so, hospital and primary care pharmacists are still relatively rare members of interprofessional teams in Norwegian clinical settings.

The Norwegian ‘Pharmacist in the Emergency Department’ (PharmED) study is designed to develop and introduce an intervention with pharmacists into the interprofessional ED team, aiming to improve medication safety for patients admitted to three Norwegian EDs [14]. Prior to the intervention, we conducted interviews with ED physicians to explore their thoughts and perceptions regarding the ED pharmacist [7]. The findings showed that ED physicians welcomed the presence of pharmacists and acknowledged a need for assistance, especially with MedRec activities. However, concerns were expressed regarding professional, structural, and legal barriers with the physician-pharmacist collaboration. The aim of this study was to explore how ED physicians experienced working with pharmacists, and their perspectives on future permanent collaboration.

## Methods

### Study design and setting

We conducted semi-structured individual interviews with ED physicians from two urban specialty care hospitals in Norway, applying a basic interpretive approach [15]. The EDs have an annual rate of approximately 16,000 (ED1) and 13,000 (ED2) admissions. The EDs are staffed with internists and surgeons affiliated with different hospital wards, working roster-based shifts. An emergency medicine specialist is present on weekdays (from 8 am to 4 pm) to supervise and assist junior (<2 years’ experience) and senior (>3 years’ experience) physicians on-call. There are two separate on-call rooms with shared workspace for internists and surgeons in the EDs. ED1 has one dedicated pharmacist desk inside the internists on-call room. However, there is limited space and number of computers available for all HCP in both EDs. In ED1, the patient’s medication chart was a printed version from the electronic health record’s medication module, and new orders were handwritten. In ED2, the electronic health record’s medication module also required updates, but the medication chart was entirely handwritten.

The pharmacist intervention began on May 3^rd^, 2021, in ED1 and on August 2^nd^, 2021, in ED2 and lasted until January 31^st^, 2022, [14]. The pharmacists were instructed to engage with patients or tasks when their expertise was sought or when they independently identified a need. Consequently, the pharmacists collaborated with the interprofessional team to establish effective ways of working on various medication-related tasks in the ED.

### National databases for medication information in Norwegian hospitals

**Fig 1:**
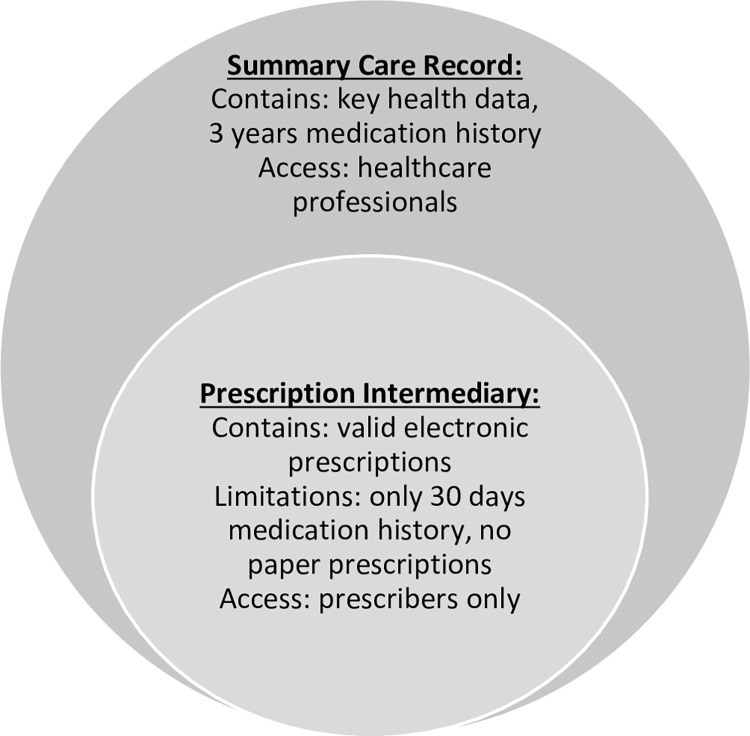
The two Norwegian electronic databases containing patients’ medication information and their main differences.

To contextualize the process of gathering medication information, perform MedRec and prescribe medications in Norwegian EDs, we here describe the two most applied and important electronically available information sources; the Summary Care Record and the Prescription Intermediary (Fig 1). The Summary Care Record is a health system that collects a selection of key health data, including all prescriptions, and can be used by HCP across health services with a read only access [16]. The Prescription Intermediary is a national central database containing patient’s valid electronic prescriptions, accessible for prescribers [17]. Prescription information can be imported from the Prescription Intermediary to electronic health records by physicians, making it a widely used information source during MedRec, and often preferred over the Summary Care Record. For patients living in nursing homes, neither the Summary Care Record nor the Prescription Intermediary can be used to obtain accurate information as they receive most of their medications from local medicine rooms (not prescriptions).

Norwegian pharmacists do not have prescribing rights, and therefore do not have the possibility to access or change information in the Prescription Intermediary, order, or sign for home medications on the medication chart after performing MedRec. Consequently, physicians must perform any necessary amendments of prescriptions themselves and sign to be responsible for orders on the medication chart even if another HCP has compiled the medication list.

### Interview guide

An interview guide (S1 File) was developed based on the following main questions; 1) What are your experiences working with pharmacists? 2) How does the collaboration between pharmacists and you work in practice? 3) After working with pharmacists in this project, what are your thoughts on working without them? The interview guide was piloted in one interview in each ED, leading to minor modifications aimed at enhancing clarity, conciseness, and ease of use, while still allowing room for discussion and follow-up questions. Informants were encouraged to share negative and positive experiences. During the interview period few negative experiences were expressed, subsequently the interview guide was adjusted in the two final interviews to specifically explore potential negative experiences with the physician-pharmacist collaboration.

### Recruitment of informants

Information about the overarching PharmED project and its sub-studies were provided in emails, closed Facebook groups, flyers, meetings, and information screens in the ED. Informants were recruited by a purposive sampling strategy aimed to maximize variation in seniority (junior or senior) and affiliation (internist or surgeon). Informants were recruited face-to-face in the ED in the morning by the main interviewer, who had no prior relationship with the informants.

All approached informants in ED1 accepted the invitation to participate and two informants in ED2 declined to participate due to lack of time and workload. The informants signed an informed consent after receiving both written and oral information about the study. They were assured that their identity would be confidential and that all personally identifiable information would be removed from the transcripts. Complete confidentiality was difficult to ensure as interviews were conducted during working hours and the physician overseeing the ED that day had to know about available physicians. All informants were informed that participation was voluntary, and they could withdraw from the study at any time before analyses had been made. During the interviews, informants were consistently encouraged to be open and honest about their experiences. Recruitment continued until the information power in our data was considered sufficient [18].

### Data collection

A main interviewer and a co-interviewer conducted the interviews in meeting rooms within or adjacent to the ED, between November 8, 2021, and January 18, 2022. We aimed for 30-45-minute interviews. Field notes were used to modify the interview guide consecutively and highlight areas to explore with other informants. No repeat interviews were conducted, and transcripts were not returned to informants for comments.

### Data analysis

Interviews were audio recorded and transcribed non-verbatim by the main interviewers, who also individually performed the preliminary analyses. Subsequently, the empirical data from the two EDs was merged and analyzed by the main author (TJ) with support from co-authors (BHG and TR). This analysis followed an inductive approach and was inspired by thematic analysis as described by Braun and Clarke [19]. The analytical process was iterative, going back and forth between the following steps; 1) Reading and re-reading data, noting initial thoughts and ideas for the analysis (TJ), discussions with co-author (TR), 2) Inductive coding using NVivo (1.7.1), accompanied by reflexive journaling during coding (TJ), 3) Collating codes and searching for themes using NVivo (1.7.1) and pen-and-paper methods (TJ), 4) Writing and reviewing themes with co-authors (TJ, BHG, TR), 5) Defining and naming both themes and sub-themes (TJ, BHG) 6) Producing final results (TJ, BHG, TR EHO, RE, EL). The preliminary analyses were also reviewed again to verify the final analysis. An example of how codes were organized into subthemes and themes can be found in S2 File.

### The research team and reflexivity

This study is a part of the PharmED study [14], where all authors are involved in the project group. The research team comprised five female pharmacists (TJ, RE, BZH, ECL, BHG), one male pharmacist (KS), one male general practitioner (TR), one male emergency medicine physician (EHO), and two female Master of Pharmacy Students (NSF, AJBT). The main interviewers (NSF, AJBT) had no previous experience in conducting qualitative interviews. However, they were trained and received feedback from their supervisors prior to and during data collection. All co-interviewers had previous experience in conducting qualitative interviews. The first author (TJ) had prior experience in conducting qualitative interviews with physicians in the same ED settings. All authors were familiar with Norwegian healthcare systems and the ED setting in which the research was conducted. The two main interviewers (NSF, AJBT) did not know the informants prior to the interviews. However, a few informants were familiar with the co-interviewers due to their work as hospital pharmacists (TJ, BZH) or as head of the ED and observation ward in ED2 (EHO).

### Ethics

Transcripts were anonymized, and informants were given a unique code and pseudonyms. All participants provided informed written consent. Quotes used in this article were translated to English by the first author (TJ) and verified by a native US English speaker with Norwegian as a second language. This research has been reported in adherence to the COREQ guidelines [20] and conducted according to ethical guidelines stated by the Helsinki declaration. The experimental protocol for the study was approved by the Data Protection Officer at Hospital Pharmacy of North Norway Trust, who serves as the Ethical Committee for the project (no. 02330).

## Results

**Fig 2:**
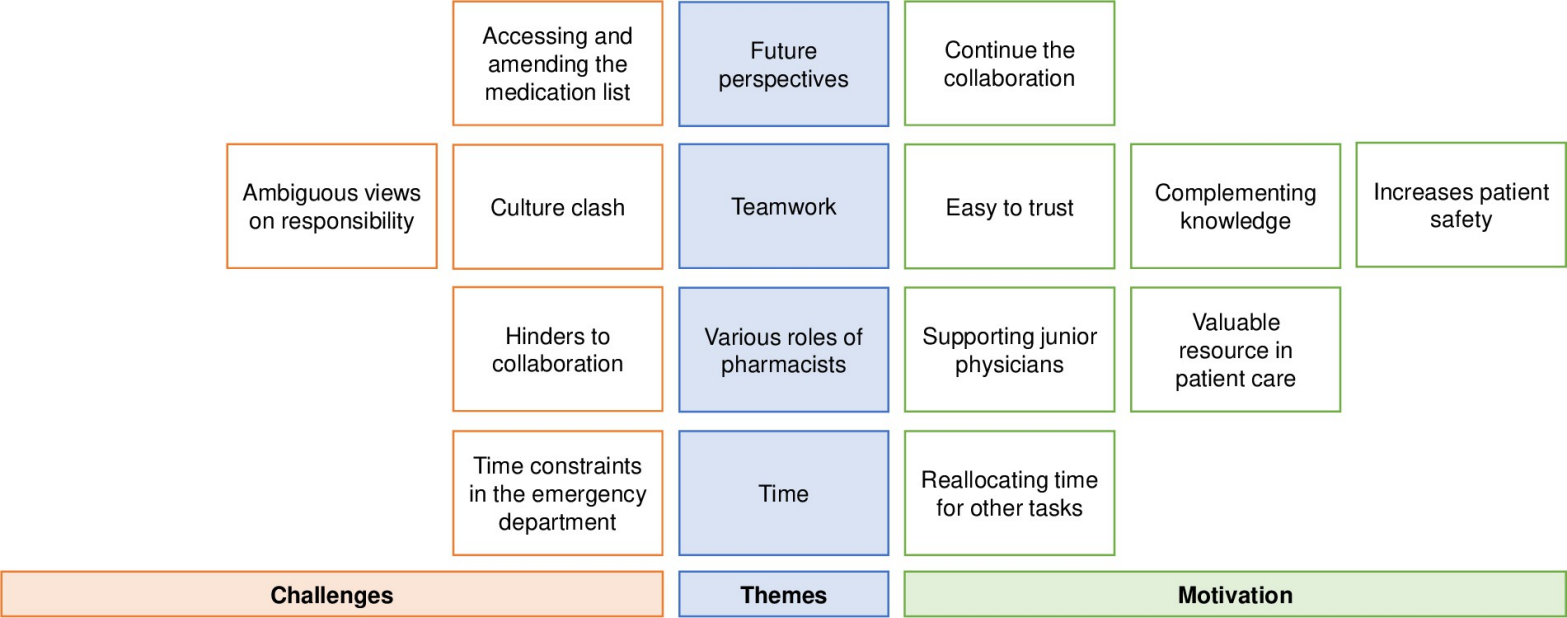
Themes and related subthemes presented as either challenges or providing motivation.

In total, 20 informants participated, with ten from each ED, comprising an equal number of ten males and ten females. Among these, six were senior internists and one was a senior surgeon, while seven were junior internists and six were junior surgeons. Interview length ranged from 16 to 37 minutes (median 28 minutes). Our final analysis identified four main themes with 12 subthemes. The subthemes are distinguished by either presenting challenges or providing motivation related to the main themes, as depicted in Fig 2, where they are positioned on opposite sides of the main themes.

### Theme 1: Time

In this theme, physicians on one hand recount their challenges in managing medication-related tasks due to time constraints in the ED, and on the other hand tell how they can reallocate time with the presence of pharmacists.

#### Time constraints in the emergency department

Informants highlighted that due to time constraints in the ED, managing patient intake and discharge often felt like a “race.” Most informants stated that in the ED, they often lacked time for MedRec tasks involving calls to home care nurses regarding patient medication use. Informants highlighted the demanding nature of the ED.

*“The emergency department is often very busy, and we have a lot of patients at once, and medication reconciliation is one of the things that demands the most time. It is very time-consuming, and consequently you often do not get to focus very much on the patient.”–* **Iselin**

Informants stressed that, in an emergency setting, their primary focus is to treat the patient’s immediate care needs, and getting a complete and accurate medication list is not their foremost concern.

*“It is very important to get it [the medication list] right, at the same time there are other things in the ED that are also very important that one may need to use more time on in a setting like that.”*–**Sigurd**

Consequently, patients could be admitted to hospital wards with inaccurate medication information and a note stating that "someone" should address MedRec later.

#### Reallocating time for other tasks

Many informants noted that collaborating with pharmacists in the ED saved physicians’ time and effort and could save them from having to work overtime.

*“For example, the patient I just had, there was a lot, and then I thought “Yes! Then your colleague [pharmacist] can fix it”. Then I can finish the paperwork, so that I am not working overtime later today if it suddenly explodes in the ED, because that happens. There is a lot of work afterwards, even though we are not in with the patient for that long.”–* **Gunnar**

Informants also mentioned that physicians could allocate more time to other patients, resulting in increased ED efficiency.

*“It is also a question about time. (…) Sometimes we spend a lot of time calling home care nurses and ask for example if the patient uses metoprolol or not. If the pharmacist is available in that situation, I need 30 minutes less to do my normal tasks.”–* **Ludvig**

### Theme 2: Various roles of pharmacists

In this theme our informants shed light on the various roles undertaken by the pharmacists during the intervention. They had positive experiences related to pharmacists’ involvement in patient care and being a support, especially for junior physicians. However, the new role also presented some challenges like e.g., availability and space constraints that hindered the collaboration.

#### Supporting junior physicians

Informants pointed out that having pharmacists particularly benefited junior physicians in the ED. Our informants emphasized that junior physicians, due to their limited experience and uncertainty in dealing with medication-related challenges, greatly appreciated the presence of ED pharmacists as a valuable resource for consultation and discussion in this setting.

*“As a new physician I have a lot to learn within the field of medications and those types of things, so it is really nice to have a professional [pharmacist] to lean on here. I think that we have had [pharmacists] less available, or we have not really had it available throughout our studies and that as well. So, I think it has been really nice to have that profession in here.”*–**Iselin**

Additionally, junior physicians are frequently tasked with conducting MedRec and therefore it eased their workload to receive help from pharmacists with MedRec. Notably, informants appreciated this especially in cases concerning patients from nursing homes, those receiving assistance from home care nurses, and individuals taking multiple medications.

*“Especially for the patients that have very long medication lists and that, it is really nice because pharmacists call home care nurses and get a completely updated list so we can be sure that the medication list is correct. (…) So, they [pharmacists] have happily taken the patients that have very complex, long medication lists, which we have a lot of in the medical department.”*–**Lan**

Given the time constraints of ED physicians, informants stressed the valuable independence of pharmacists in patient care. The pharmacists frequently initiated MedRec prior to physician recommendations, earning praise for their proactive approach.

#### Valuable resource in patient care

In addition to receiving help with MedRec, our informants highlighted pharmacists’ involvement in reviewing medications and as being valuable resources for physicians to address medication-related issues.

*“My experience [with pharmacists] has been overwhelmingly positive and I think it has been very nice to be able to have professional discussions with pharmacists as well. Not only receiving a finished medication list, which is of course very convenient when admitting patients, but also the medication review process as well. I think that has also been very nice.”*–**Henrik**

Informants found medication recommendations from pharmacists valuable. Examples of such advice included to assess interactions, adjust medication timing, deprescribe, dose antibiotics, address improper medication use, select appropriate analgesics, and how to manage adverse effects. While some informants occasionally disagreed with the advice given, they still valued the pharmacist’s input, deeming it important to consider.

*“They [pharmacists] talk to patients, they review medications, they give advice on medications we should stop, switch, change dose, and if there is anything we should follow up with a serum drug concentration, those types of things. Really, it is very nice.”*–**Lan**

#### Hinders to collaboration

The informants expressed that for varied reasons the pharmacist was not always available to help physicians. Firstly, the pharmacist could be occupied with another patient, often with a long medication list which takes time to complete. Secondly, because the internists and surgeons shared the pharmacist, and thirdly because of their working hours since pharmacist’s shift ended at 7 pm. A few informants also expressed that if the pharmacist were not present in the on-call room, physicians could forget about the possibility to receive help from the pharmacist.

*“Occasionally the pharmacists are busy with another patient. And then there ends up not being enough resources available.”–* **Robert**

Three informants also expressed that a negative side to receiving help from pharmacists was that you as physician could sometimes lose overview of the patients’ medications when you do not do all the work yourself.

*“But what is negative with it is that I feel like I have a little less ownership and possibly a little less control. I have to make sure that I look over and assess the medication list before I admit the patient.”–* **Kari**

Most informants from both EDs pointed out that a downside of having pharmacists was the lack of room, space, and computers. Informants noted that this issue existed before ED pharmacists were introduced and was not the fault of the pharmacists. Even so, this often meant that someone, usually a pharmacist, junior physician, or medical student, had to work at nurse’s stations or in the break room. Informants acknowledged the space constraints, saying:

“*That small room does not have enough space for as many people that are in there really, (…) but it is not the pharmacist’s fault. It is the ones who have allocated such a small on-call room for so many people, because medical students are also supposed to have the opportunity to be there, but it is really not enough space.”–***Henrik**

Informants also emphasized the importance of having pharmacists present in the ED for the sake of collaboration and expressed a preference for face-to-face communication over phone calls. Consequently, they expressed a need for sufficient workspace so they could work together in the same room.

*“I think that there needs to be space to work together somewhere, for example a shared office or more space in the examination rooms. Things will work even better then. Because if we share an office, often times when you sit and work together, then you get asked “are you thinking about this” etc. Or if you are working together in the examination room, and you hear each other talk to the patient, right? I think that would be a dream scenario, to have everyone in the same space.”–* **Elisabeth**

### Theme 3: Teamwork

This theme delves into distinctive characteristics that are important when working in a team. On one hand, our informants experienced that it was easy to trust pharmacists, that pharmacists’ complementing knowledge provided new perspectives in a team, and that teamwork increased patient safety. On the other hand, our informants shared some experiences with ambiguous views on responsibility and found that physician-pharmacist collaboration could depict a culture clash, both presenting potential challenges within a team.

#### Easy to trust

All informants expressed that they trust the pharmacists, and for some this trust developed during teamwork. Many informants also expressed an increasing confidence in the accuracy of medication lists following the involvement of pharmacists. Having witnessed their meticulous work, most informants indicated a higher level of trust in pharmacists’ ability to perform MedRec.

*“I have no problem trusting pharmacists. I trust a lot of other people that I have even less of a reason to trust than a pharmacist who has reconciled a medication list.”*–**Ahmed**

Only physicians can sign off the reconciled medication list, hence physicians must trust the ED pharmacist implicitly, or double check the reconciled medication list. Several physicians emphasized their readiness to assume responsibility for pharmacists, citing their thoroughness, electronic health record documentation, accountability, and the fact that pharmacists had initially taught them about MedRec as reasons for their confidence.

#### Ambiguous views on responsibility

Even though our informants trusted pharmacists, they at the same time had ambiguous views regarding taking responsibility for MedRec performed by pharmacists. Some informants felt that the person performing MedRec should be responsible for signing the medication chart with the reconciled medication list. This viewpoint arose from physicians’ reluctance to assume responsibility for potential mistakes made by others. Some informants believed in a shared responsibility approach, while others believed that the focus should be on appreciating the assistance provided by pharmacists rather than dwelling on responsibility.

*“I think that as a physician you have the overall medical responsibility of course, but to delegate is a part of the job. And pharmacists write notes about what they do, and we write in the admission note that it is a pharmacist who has reconciled the medication list. Of course, the list is printed and signed by us, but they also have a responsibility to ensure that it is correct. So, we are a team […] so I do not see a problem with it.”–* **Sigurd**

Some informants still felt that the ultimate responsibility rests with physicians because they sign the medication chart, thereby affirming its correctness. Consequently, a few informants checked the medication lists provided by pharmacists. Many informants considered assuming responsibility for various tasks as a natural part of their daily work. One informant gave an example of how this encompassed a broad spectrum of tasks, i.e., from medications to transportation decisions. Taking responsibility for the work of others was seen as part of the hierarchical structure they operated within, with junior physicians overseeing work by medical students and pharmacists, and senior physicians supervising junior physicians.

*“There is always a risk involved when taking responsibility for others [work], but we do it on a daily basis. The physician supervising me takes responsibility for all measures initiated by junior physicians, and I take responsibility for pharmacists, so that is completely normal. I have no concerns in taking responsibility for that […]. I think that the pharmacy education here is good, so that works out just fine.”*–**Ludvig**

#### Culture clash

Many informants used descriptors like *thorough* and *accurate* when characterizing pharmacists and said pharmacists had an elevated level of detail in their work. We identified that physicians believe that pharmacists and physicians have different perspectives, approaches, and attitudes about the importance of medications. One informant encapsulated this by describing the collaboration between physicians and pharmacists as a culture clash.

*In the world of physicians, things tend to go, it goes like “chop, chop, chop” [mimics with hands], it often goes a little fast, and it stays on a surface level. You [physicians] fix what is important, and most of it will work itself out afterwards. And then the pharmacists arrive and go through everything in detail, and you do not need all these details. (…) They [pharmacists] spend a lot of time and energy fixing everything very good, very thoroughly and very perfect, and in reality, no one is interested in having it that perfect. (…) So, it is little bit of a culture clash on that, I think.”*–**Lotte**

A few informants also said that pharmacists occasionally raised questions they deemed irrelevant, especially in the fast-paced ED environment.

#### Complementing knowledge

Many informants believed the pharmacists brought fresh perspectives to medication matters the physicians had not considered. This contribution was highly valued in the interprofessional team, as it involved two HCP reviewing medication regimens, each with a unique approach.

*“And if you think of society as a whole, there are many [patients] that come to EDs with adverse effects from medications, which you can catch earlier if you have pharmacists there at all times.”*–**Mona**

A few informants mentioned that pharmacists assisted in identifying potential reasons for hospitalization from perspectives physicians had not considered. They highlighted that the pharmacists consistently provided relevant comments, emphasizing the complementary nature of the knowledge between physicians and pharmacists.

*“There are things that the others and I have not thought about. Or I have never thought about it because I do not really think as much about the medication list if there are not any questions about it. But I understand that there are relevant issues, and I think it is very important that in a way they are brought to light. Or how do you put it, that at least it gets pointed out.”–* **Ali**

Both junior and senior physicians expressed that they learned a lot from pharmacists and vice versa. They believed that every HCP had a unique role in the ED, and the collaboration served the patient’s best interests.

*“Yes, we learn from each other all the time, you are a team. So, everyone has their own role, and everyone learns from each other. You guys [pharmacists] have completed an education we have not. So, we know about medications, although not as much, right? We have a lot of other things to learn during our studies. So, it is nice to have each other. The same as with nurses, physicians, pharmacists, we work in a team (…) and we each play a very important role no matter who we are.”–* **Elisabeth**

What informants expressed they learned from pharmacists ranged from technical aspects of MedRec and medication management to interactions, antibiotics, and dosing in patients with impaired renal function, among many other topics.

#### Increases patient safety

*“Like, it increases the quality. It decreases the chance of medication errors. (…) and your question was what does that mean for the patient? It is quite frankly a quality assurance, I think. And it has happened many times during my shift, that there have been errors in the medication list at admission. We have given twice the dose, tenfold the dose, right? It happens. And it [having pharmacists] decreases… I am sure it decreases the risk of that. Guaranteed.”–* **Stian**

Our informants had several reasons why working with pharmacists was beneficial for patients. Firstly, pharmacists could focus solely on medication-related tasks, unlike physicians who had numerous responsibilities. Informants stressed that medication-related tasks required undivided attention, which physicians could not always provide. Secondly, pharmacists were meticulous in their work, with some informants noting that they interacted with patients in a more organized and comprehensive manner. Thirdly, pharmacists had the time to contact other sources during MedRec to obtain accurate medication lists, such as home care nurses or pharmacies. Therefore, having pharmacists were seen as a quality assurance to prevent further errors down the line. A final explanation for the increased patient safety was having both pharmacists and physicians assess medications from different perspectives.

*“And also, the part with having two sets of eyes too. One thing is the medication reconciliation, but you are also two sets of eyes that look at the same medication list and can also get input [from pharmacists] if there are any strange things there. And that means you pay extra attention in a team.”*–**Emil**

### Theme 4: Future perspectives

This theme concerns future perspectives of the interprofessional collaboration with ED pharmacists. All informants wanted the physician-pharmacist collaboration to continue after the project period. However, our informants saw an improvement potential in providing pharmacists with the opportunity to perform the entire MedRec procedure independent of physicians.

#### Accessing and amending the medication list

Most informants described in a positive and grateful way that pharmacists provided a “recipe” for physicians to follow when updating the Prescription Intermediary as a part of the MedRec procedure. However, this was sometimes perceived as physicians having to do clean-up work and a few informants identified pharmacists’ lack of access to the Prescription Intermediary as a problem with a potential of improvement.

*“What I see as an issue is that the pharmacist does not have access to the prescription intermediary, so to sum up, it basically does not go any faster. That is probably in a way what is not living up to the expectations, but that does not have anything to do with the people or the pharmacists. It has to do with system access. (…) I think that if they [pharmacists] had access to the prescription intermediary, it would be less back and forth, here and there. Then it would be like, ok, here is a complete MedRec and chart, and then the treating physician could review the medications and sign for it. I think that is the biggest room for improvement.”*–**Lotte**

While informants acknowledged that the current approach was better than receiving no assistance at all, they believed there was potential to enhance the workflow and save time in this area.

#### Continue the collaboration

Most informants wanted to continue having pharmacists in the ED, and when considering the employment of pharmacists in the ED versus other hospital wards, informants assessed pharmacists’ contributions differently in those settings. Many emphasized the importance of MedRec in the ED, while suggesting that a more comprehensive medication review might be better suited for a ward setting. While having pharmacists in the ED was seen as a clear advantage, many medication-related questions required follow-up later and one informant saw a need to better determine this workflow.

*"But I actually think that it’s useful to have a pharmacist in the ED, but then I think about the next step, and it should be a prerequisite to place responsibility for follow-up questions so that they [pharmacists] can be utilized to the best extent."–* **Anne**

Some informants found it unreasonable having to choose between an ED pharmacist and a ward pharmacist, as they preferably would have both. However, nearly all informants favored retaining ED pharmacists post-project if they had to choose. Many informants also indicated a desire for an increased number of pharmacists within the ED team, as well as their assistance during night shifts. One informant highlighted that patients get sick beyond regular working hours, emphasizing the need for 24/7 pharmacist availability.

*“Yeah, I think it is very smart having you [pharmacists] here. Actually, we need more pharmacists. I am not sure how you are currently distributed, because I am not up to speed with that. But anyways, the surgical side needs one, and the medical side needs two pharmacists. Because there is so much to do (…) and the medical side often has more complicated patients, often with a greater need for a medication review.”*–**Elisabeth**

Additionally, many informants expressed worry about the prospect of managing MedRec for all patients themselves post-project. One informant humorously extended a "good luck" to the new junior physicians who would have to work in an ED without pharmacists.

## Discussion

In this study, four themes (*time*, *various roles of pharmacists*, *teamwork*, and *future perpectives*) were identified that describe how ED physicians experience collaborating with pharmacists in their daily work during an intervention study, and their future perspectives on a permanent collaboration with pharmacists. These four themes were further divided into twelve subthemes, distinguished by presenting challenges or providing motivation related to the main themes. While there is a generally positive attitude towards having pharmacists as a permanent part of the interprofessional ED team, our results also highlights challenges that requires attention and solution.

When reflecting on medication-related tasks, our informants focused on MedRec, which is consistent with findings prior the intervention [7]. They reiterated the time-consuming nature of MedRec, underscoring that obtaining a comprehensive medication list in the fast-paced ED environment could not always be prioritized due to the time constraints. This aligns with the findings by Boockvar *et al*., who identified that when time is limited, other responsibilities are prioritized over MedRec [21]. Our informants highlighted that receiving assistance from pharmacists with medication-related tasks significantly eased their workload. They reported having more time to perform other tasks while entrusting pharmacists with responsibilities such as MedRec. Task sharing or task shifting, as proposed in the Norwegian 2023 Health Personnel Commission’s report titled ‘Time to act’ [22], is considered one of the strategies to address the increasing pressure on HCP. Norway, like many other countries, faces significant challenges related to HCP availability [22]. The task sharing or task shifting approach underlines collaborative efforts to maximize expertise and capabilities effectively. Our results show that pharmacists performing MedRec is highly desired by physicians. In addition to conducting MedRec, our results show how pharmacists complemented physicians’ knowledge and provided valuable medication-related recommendations. This result aligns with findings by Mogensen *et al*., who identified that ED pharmacists detected serious medication-related problems that had not been identified by physicians before [23]. This emphasizes the importance of diverse competencies in a team. Utilizing the role of pharmacists in healthcare services is recommended by the World Health Organization, stating that pharmacists are suitable for task shifting in health care due to their knowledge of medicines and clinical therapeutics [24]. In US and Canada, the ED pharmacist role has expanded over several decades, transitioning from medication distribution to include direct patient care services [25–27]. ED pharmacist activities encompass clinical tasks, emergency response, MedRec/medication history taking, and teaching [25–27]. Their contributions are highly valued within the interprofessional team [28]. It was a clear perception among our informants that the physician-pharmacist collaboration in the ED should be established as a permanent practice. The establishment of professional trust in pharmacists was surprisingly uncomplicated and not perceived as particularly challenging. Nevertheless, our informants expressed ambiguities regarding their responsibility for tasks conducted by pharmacists. Many suggested that pharmacists should be provided access to update the medication list in the prescription intermediary/electronic health records, thereby alleviating some of the physicians’ workload instead of adding extra. According to Nancarrow *et al*., essential characteristics of a good interprofessional team include ensuring appropriate resources and procedures, in addition to respecting and understanding roles [29]. Addressing the challenges related to roles and responsibilities in Norwegian EDs may necessitate the involvement of Norwegian authorities and change of legal rights for pharmacists. This is crucial for enhancing the efficiency of the interprofessional collaboration between physicians and pharmacists in EDs. Space constraints in the ED was expressed as a hinder to effective interprofessional collaboration. This challenge was also identified prior to the intervention [7]. Nancarrow’s ten characteristics for good interprofessional teams, include team members working from the same location [29], a condition echoed by our informants who expressed a desire for sufficient space to work together and communicate face-to-face. Other studies support this perspective; Coralic *et al*. found that having pharmacists present in the ED also increased the likelihood of consultation [30]. Brewer *et al*. highlighted the significance of a dedicated space for collaboration and learning in interprofessional education, emphasizing that building personal relationships through informal and social communication formes the basis for effective interprofessional working relationships [31]. The importance of place and space for interprofessional communication and collaboration is further supported by Oandasan *et al*. and Kitto *et al*. [32, 33]. In summary, our findings underpins the necessity of providing interprofessional teams in EDs and other settings with adequate space to enhance effective collaboration and interaction.

### Strengths and limitations

The main strength of the study lies in the recruitment of a diverse sample of physicians from two ED settings, enhancing the study’s information power [18]. Our informants possessed firsthand knowledge about the phenomenon of interest, and the final analysis revealed similar perceptions and experiences of working with ED pharmacists in both EDs. Consequently, we believe our results may be representative to the physician-pharmacist collaboration in other ED settings. Another strength is that findings in the final analysis were supported by the two preliminary analyses, further strengthening the validity of our results. Finally, the analysis was conducted with the support of an interprofessional team, enabling a multifaceted examination of the results from different interprofessional perspectives.

However, the results also have limitations. First, there is a potential information bias, as we may have interviewed only physicians who had positive views towards the ED pharmacist collaboration. Consequently, we may have missed information that could have provided deeper insights into the phenomenon of interest. Second, both interviews and analyses may have been influenced by researchers with pharmacy background. Third, the informants associated the researchers with ED pharmacists during interviews, even if none of the researchers worked in the ED. This association may have induced hesitancy towards speaking negatively about another profession or colleague, potentially resulting in overly positive results regarding the ED pharmacist.

## Conclusion

Physicians’ experiences with the collaboration with pharmacists in the ED suggests that pharmacists should be permanently integrated into the interprofessional ED team. Their contributions to MedRec and knowledgeable medication-related recommendations have led to a perceived increase in ED efficiency and patient safety. However, certain barriers to collaboration should be addressed to further enhance interprofessional teamwork. Authorities should recognize and leverage pharmacists’ knowledge and competencies, taking appropriate steps empower pharmacists to compile medication lists in EDs. Additionally, it is important to ensure sufficient physical space for the ED teams.

## Supporting information

S1 File Interview guide. (DOCX)

S2 File Organization of data material to theme example. (DOCX)
