# Supplementary material for: Physicians’ experiences with pharmacists as new members of the interprofessional emergency department team. A qualitative study
Source: PLoS One. 2025 Jan 13;20(1):e0317298. doi: 10.1371/journal.pone.0317298 (PMC11729929; doi:10.1371/journal.pone.0317298)
Supplement: S1 File — (DOCX) [file pone.0317298.s001.docx]

# Supplement 1

**Interview guide**

Opening question:

The ED pharmacists started working in the ED May 3^rd^/August 2^nd^, and you have worked together for X (fill in) months now. How has the time with ED pharmacists been for you?

Further questions:

1. Do you have previous experience of working with pharmacists at hospital wards? And if yes, how do you experience the collaboration in the ED vs. the ward?
2. How have working with ED pharmacists affected your day?
3. How do you proceed when you want to communicate with the pharmacists? Are they available when you need them?
4. Which work tasks do ED pharmacists have?
5. ED pharmacists do not work 24/7. How have the pharmacists influenced your way of working?
6. How does the pharmacists’ way of working *work* in an ED setting?
7. What do you feel that you need help with (regarding medications) in the ED?
8. Have you learned something by working with ED pharmacists?
9. The project end is closing in, what are your thoughts on this?
