# Supplementary material for: Physicians’ experiences with pharmacists as new members of the interprofessional emergency department team. A qualitative study
Source: PLoS One. 2025 Jan 13;20(1):e0317298. doi: 10.1371/journal.pone.0317298 (PMC11729929; doi:10.1371/journal.pone.0317298)
Supplement: S2 File — (DOCX) [file pone.0317298.s002.docx]

# Supplement 2

This example shows how data material was coded and organized into subthemes and themes. The table contains a selection of data extracts and codes.

| Data extract | Code | Subtheme | Theme |
| --- | --- | --- | --- |
| It is not always we physicians necessarily have the time or opportunity to do it [medication reconciliation] in the emergency department. | Physicians can not prioritize medication reconciliation | **Time constraints in the emergency department** | **Time** |
| We do our best with regards to medication reconciliation, but it is not always, if the patients do not know themselves which medications they use and there are 20 patients in the emergency department, it says itself that we do not have the time to call home care nurses and ask. | Medication reconciliation is time-consuming, and time is a precious asset in a crowded emergency department |  |  |
| The emergency department is often very busy, and we have a lot of patients at once, and medication reconciliation is one of the things that demands the most time. It is very time-consuming, and consequently you often don’t get to focus very much on the patient | Busy emergency department and time-consuming tasks like medication reconciliation steals focus from the patient |  |  |
| It happens that patients are hospitalized without the medication list being reconciled if the emergency department is very busy | Patients are hospitalized with inaccurate information about medications due to time constraints |  |  |
| I have to admit that I do not take the time to do medication reconciliation, I have just quit with it because I believe I have so many other tasks that I do not want the responsibility for it, and I think someone else has to do it in daytime. | Other tasks have a higher priority due to limited time, and medication reconciliation can be postponed to daytime |  |  |
| Major time saving! (…) You save a lot of time [having pharmacists conduct medication reconciliation] | Pharmacists in emergency departments saves time for physicians | **Reallocating time for other tasks** |  |
| It lets me become more efficient, with more patients. You can assess patients faster when you receive assistance. | Assistance from pharmacists makes physicians more efficient in managing more patients |  |  |
| Simply put, it is much less work. | Less work for physicians with the assistance of pharmacists |  |  |
| You get more physician resources available, which can be used with acute ill patients | More time for physicians to focus on acute ill patients |  |  |
| For example, the patient I had earlier, it was a lot, and then I thought “Yes! Your colleague [pharmacist] can fix that.” Then I could complete the paperwork, so I do not have to work overtime later today, if it suddenly “explodes” in the emergency room, because that happens. | Time physicians save by having pharmacists conduct medication reconciliation can be used on other tasks, which can prevent overtime if the emergency room suddenly becomes crowded |  |  |
| If the pharmacist is present in that situation, I need 30 minutes less to do my normal tasks. | Physicians need 30 minutes less to complete their normal tasks with presence of pharmacists |  |  |
